# Folate intake, serum folate levels, and prostate cancer risk: a meta-analysis of prospective studies

**DOI:** 10.1186/1471-2458-14-1326

**Published:** 2014-12-29

**Authors:** Rong Wang, Yan Zheng, Jing-Yang Huang, Ai-Qin Zhang, Yu-Hao Zhou, Jie-Ning Wang

**Affiliations:** Department of Urinary Surgery, Shanghai Seventh People’s Hospital, Shanghai, China; Department of Science, Shanghai Seventh People’s Hospital, Shanghai, China; Department of Nursing, Shanghai Seventh People’s Hospital, Shanghai, China; Department of Rehabilitation Institute, Shanghai Seventh People’s Hospital, Shanghai, China; Shanghai Seventh People’s Hospital, Shanghai, China

**Keywords:** Folate, Prostate cancer, Dose–response, Meta-analysis

## Abstract

**Background:**

Studies have reported inconsistent results concerning the existence of associations of folate intake and serum folate levels with prostate cancer risk. This study sought to summarise the evidence regarding these relationships using a dose–response meta-analysis approach.

**Methods:**

In January 2014, we performed electronic searches of PubMed, Embase, and the Cochrane Library to identify studies examining the effect of folate on the incidence of prostate cancer. Only prospective studies that reported effect estimates with 95% confidence intervals (CIs) of the incidence of prostate cancer for more than 2 categories of folate were included.

**Results:**

Overall, we included 10 prospective studies reporting data on 202,517 individuals. High dietary folate intake had little or no effect on prostate cancer risk (risk ratio [RR] = 1.02; 95% CI = 0.95–1.09; P = 0.598). The dose–response meta-analysis suggested that a 100 μg per day increase in dietary folate intake has no significant effect on the risk of prostate cancer (RR = 1.01; 95% CI = 0.99–1.02; P = 0.433). However, high serum folate levels were associated with an increased risk of prostate cancer (RR = 1.21; 95% CI = 1.05–1.39; P = 0.008). The dose–response meta-analysis indicated that a 5 nmol/L increment of serum folate levels was also associated with an increased risk of prostate cancer (RR = 1.04; 95% CI = 1.00–1.07; P = 0.042).

**Conclusions:**

Our study indicated that dietary folate intake had little or no effect on prostate cancer risk. However, increased serum folate levels have potentially harmful effects on the risk of prostate cancer.

**Electronic supplementary material:**

The online version of this article (doi:10.1186/1471-2458-14-1326) contains supplementary material, which is available to authorized users.

## Background

Prostate cancer is the second most common non-skin cancer among men globally, and with markedly higher incidence rates in developed countries [1, 2]. Over the past few decades, studies [3, 4] have revealed that age, ethnicity, and family history could influence the incidence of prostate cancer, and migrant studies suggested that a healthy diet and lifestyle are critical for preventing prostate cancer [5]. Folate has long been hypothesised to be related to cancer risk [6]. However, data on the effect of dietary folate intake or serum folate levels on subsequent prostate cancer morbidity are limited and inconclusive.

The results of a previous prospective study [7] indicated that dietary folate intake was associated with a greater risk of prostate cancer at a certain dose. Several other prospective studies [8–16] revealed that folate intake has little or no effect on the risk of prostate cancer. Clarifying the optimal folate levels in the general population is particularly important, as these values have not been definitively determined. Traditional case control studies are sensitive to confounding factors and bias, especially recall bias.

In 2012, Wien [17] used a standardised approach to review the available evidence, and concluded that folate intake increased the risk of prostate cancer by 24% when compared with participants received placebo. However, additional unanswered questions remain, such as whether these effects differ in several specific subpopulations. A collaborative analysis of observational studies [18] showed a 43% increase in the risk of prostate cancer with a high serum folate level. However, the dietary or serum folate category cut-off values differ among included studies. Furthermore, previous meta-analysis is the inclusion of retrospective case–control studies, which are sensitive to confounding factors and bias, especially recall bias.

In this study, we attempted a large-scale examination of the available prospective studies to determine the association between dietary folate intake or serum folate levels and prostate morbidity. We also performed a dose–response meta-analysis to quantify the risk of prostate cancer associated with incremental increases in dietary folate intake and serum folate levels for the general population.

## Methods

### Data sources, search strategy, and selection criteria

This review was conducted and reported according to the Preferred Reporting Items for Systematic Reviews and Meta-Analysis Statement issued in 2009 (Additional file 1: Checklist S1).

We systematically searched the PubMed, Embase, and Cochrane Library electronic databases (from database inception to Jan 2014), with no language restrictions for studies in humans. We included all studies investigating an association between dietary folate intake or serum folate levels and prostate cancer incidence. Our core search included the following terms: (“folate” OR “folic acid”) AND (“prostate cancer” OR “prostate neoplasm” OR “prostate carcinoma”) AND (“cohort” OR “cohort studies” OR “nest case–control studies”). If a site-specific dataset had been published more than once, we used the most recent publication. We reviewed the reference lists of the identified reports, reviews, meta-analyses, and other relevant publications to find additional pertinent studies. The medical subject heading, methods, population, study design, exposure, and outcome variables of these articles were used to identify relevant studies.

A study was eligible for inclusion if the following criteria were met: (1) the study had a prospective observational design (prospective cohort or prospective nested case–control study); (2) the study investigated the association between dietary folate intake or serum folate levels and the risk of prostate cancer; and (3) the authors reported effect estimates (risk ratio [RR], hazard ratio [HR], or odds ratio [OR]) and 95% confidence intervals (CIs) for comparisons between high and low dietary folate intake or serum folate levels (with more than 2 categories). We excluded all retrospective case–control studies because various confounding factors could bias the results.

The literature search was independently undertaken by 2 authors (RW and YZ) using a standardised approach. Any inconsistencies were resolved by discussion with the primary author (YHZ) and a consensus was reached.

### Data collection and quality assessment

The following data elements were collected: name of the first author or study group, publication year, country, study design, assessment of folate levels, sample size, age at baseline, effect estimate, comparison categories, follow-up duration, and covariates in the fully adjusted model. We also extracted the numbers of cases per person or per person-year, effect of the different exposed categories, and 95% CIs. For studies that reported several multivariable adjusted RRs, we selected the effect estimate that was maximally adjusted for potential confounders.

The Newcastle-Ottawa Scale (NOS) was used to evaluate methodological quality [19, 20]. The NOS is a comprehensive tool that has been partially validated for evaluating the quality of observational studies in meta-analyses [19]. The NOS is based on the following 3 subscales: selection (4 items), comparability (1 item), and outcome (3 items). A “star system” (range, 0–9) has been developed for assessment (Additional file 2: Table S1). The data extraction and quality assessment were conducted independently by 2 authors (YZ and JYH). Information was examined and adjudicated independently by an additional author (JNW), who referred to the original studies.

### Statistical analysis

We examined the relationship between dietary folate intake or serum folate levels and the risk of prostate cancer based on the effect estimate (OR, RR, or HR) and its 95% CI published in each study. We first used the random-effects model to calculate summary RRs and 95% CIs for the highest folate levels compared to the lowest folate levels [21, 22]. We subsequently transformed category-specific risk estimates into estimates of the RR associated with every 100 μg per day increase in dietary folate intake and every 5 nmol/L increase in serum folate levels via the generalised least squares for trend estimation. These estimates were calculated from the assumption of a linear relation between the natural logarithm of the RR and increasing folate levels [23, 24]. The value assigned to each folate level category was the mid-point for closed categories and the median for open categories (assuming a normal distribution for folate levels) [23]. We combined the RRs for each 100 μg per day increase in dietary folate intake and each 5 nmol/L increase in serum folate levels via a random-effect meta-analysis. Unless otherwise stated, we used the most adjusted risk estimate from each study as stated previously. We finally conducted a dose response random-effects meta-analysis from the correlated natural log of RRs or HRs across the folate levels categories. To derive the dose–response curve, we modelled folate using restricted cubic splines with 3 knots at fixed percentiles of 10, 50, and 90% of the distribution [23]. This method requires the effect measure with its variance estimate for at least 3 known categories of exposure. We assessed the heterogeneity between studies using the I^2^ statistic to measure the proportion of total estimate variation that was attributable to study heterogeneity; I^2^ values of 25%, 50%, and 75% were used as cut-off points for low, moderate, and high degrees of heterogeneity, respectively [25, 26]. Subgroup analyses were conducted for prostate cancer based on country, effect estimate, duration of follow-up, and adjustment for age, BMI, alcohol consumption or smoking status. We also performed a sensitivity analysis by removing each individual study from the meta-analysis [27]. Several methods were used to assess potential publication bias. Visual inspections of funnel plots for prostate cancer were conducted. The Egger [28] and Begg tests [29] were also used to statistically assess publication bias for prostate cancer. All reported P values were 2-sided, and P values <0.05 were considered statistically significant for all included studies. Statistical analyses were performed using STATA software (version 12.0; Stata Corporation, College Station, TX, USA).

## Results

**Figure 1:**
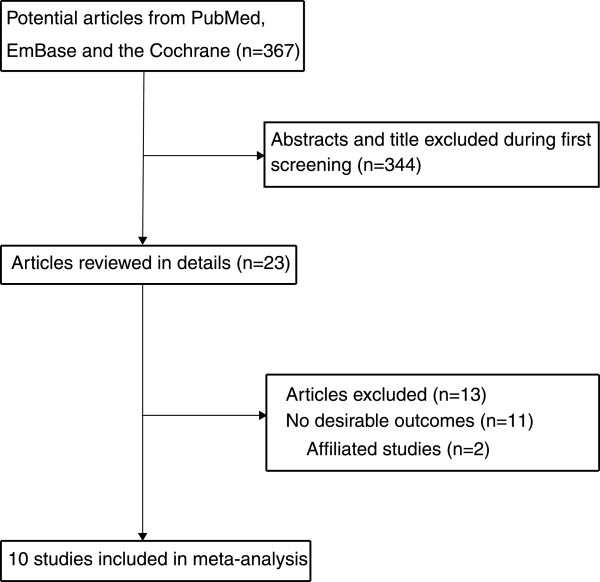
**Flow diagram of the literature search and studies selection process.**

The results of the study selection process are shown in Figure 1. We identified 367 articles in our initial electronic search; 23 remained after exclusion of duplicates and irrelevant studies. After a detailed evaluation, 10 prospective studies [7–16] were selected for the final meta-analysis. A manual search of the reference lists of these studies did not yield any new eligible studies. The general characteristics of the included studies are presented in Table 1.

**Table 1:** Baseline characteristic of studies included in the systematic review and meta-analysis

Study	Country	Study design	Assessment of exposure	Sample size	Age at baseline	Effect estimate	Comparison categories	Follow-up (year)	Covariates in fully adjusted model
SJ Weinstein^8^ 2006	Finland	Cohort	FFQ	27111	50-69	RR	>378 μg/d versus <283 μg/d	12.4	Age, vitamin supplement use, energy intake
VL Stevens^9^ 2006	US	Cohort	FFQ	65836	50-74	RR	>347 μg/d versus <204 μg/d	9.0	Age, race, education, total calories, total calcium, ethanol, family history of prostate cancer, vitamin B12, prostate-specific antigen screening, and history of diabetes
JK Bassett^10^ 2012	Australia	Cohort	FFQ	14620	40-69	HR	444 μg/d versus 215 μg/d	15.0	Country of birth, education, alcohol consumption, BMI, and daily intakes of lycopene and calcium
BAJ Verhage^11^ 2012	Netherland	Cohort	FFQ	58279	55-69	HR	>259.1 μg/d versus <176.5 μg/d	17.3	Age
N Roswall^12^ 2013	Denmark	Cohort	Self-administer questionnaire	26856	50-64	HR	>412.9 μg/d versus <280.5 μg/d	17.0	Intake of the three other micronutrients as well as dietary intake for the supplemental intake and supplemental intake for the dietary intake and further for height, weight, education, intake of red meat, alcohol consumption, selenium intake
M Johansson^13^ 2008	Europe	Nest case control	Blood samples	2043	58.7	RR	>16.55 nmol/L versus <4.82 nmol/L	5.0	Body mass index, smoking status, alcohol intake, physical activity, marital status, and education level
J Beilby^14^ 2010	Australia	Nest case control	Blood samples	321	69.5	OR	Tertiles 3 versus tertiles 1	6.0	Age, administered vitamin A supplement
S Vogel^15^ 2013	Norway	Nest case control	Blood samples	6000	49.1	OR	>17.5 nmol/L versus <10.9 nmol/L	15.7	Serum creatinine concentration, education, smoking, physical activity and body mass index.
J Hultdin^7^ 2005	Sweden	Nest case control	Blood samples	768	58.2	OR	>10.3 nmol/L versus <5.85 nmol/L	4.9	Other 2 plasma variables, BMI and smoking
SJ Weinstein^16^ 2003	Finland	Nest case control	Blood samples	678	50-69	OR	>10.79 nmol/L versus <6.87 nmol/L	6-9	Benign prostate hyperplasia

Five prospective cohort studies [8–12] involving a total of 192,702 individuals, between 14,620 and 65,836 men were included in each study, and follow-up periods ranged from 9.0 to 17.3 years evaluated the association between dietary folate intake and the risk of prostate cancer, and the remaining 5 nested case control studies [7, 13–16] investigated the association between serum folate levels and the risk of prostate cancer for a total of 9810 individuals, between 321 and 6000 blood samples were included in each study, and follow-up periods ranged from 4.9 to 15.7 years. Furthermore, serum folate level was measured on a non-fasting sample obtained at entry to the study in all included studies. One study [9] was conducted in the United States, 2 [10, 14] were performed in Australia, and the remaining 7 studies [7,8,11-13.15,16] were conducted in Europe. Study quality was assessed using the NOS system [19]. In this study, we considered a study with a score of 8 or 9 as being of high quality. Overall, three studies [8, 10, 15] had a score of 9, four studies [7, 9, 11, 12] had a score of 8, two studies [13, 16] had a score of 7, and one study [14] had a score of 6.

### Dietary folate intake and the risk of prostate cancer

**Figure 2:**
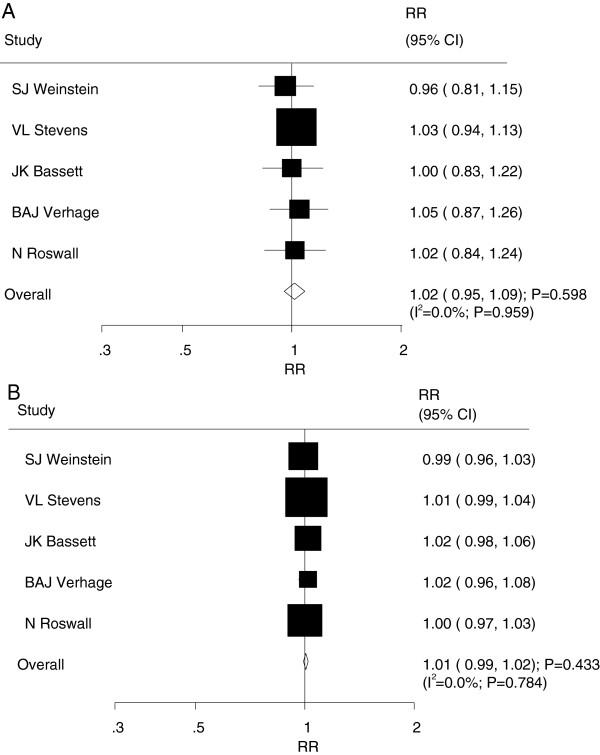
**Relative risk estimates of prostate cancer for high versus low dietary folate intake (A) and per 100 ug/day increment in folate intake for prostate cancer (B).**

**Figure 3:**
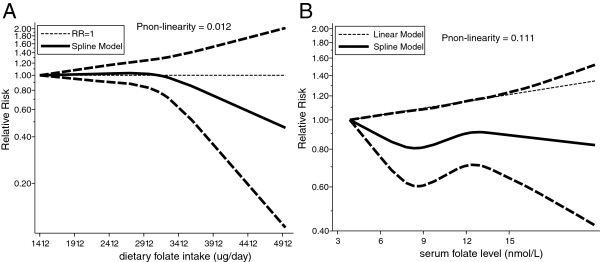
**Dose-response relations for dietary folate intake (A) and serum folate levels (B).**

After pooling the included studies [8–12], the summary RR illustrated that a high dietary folate intake was not associated with prostate cancer risk (RR = 1.02; 95% CI = 0.95–1.09; P = 0.598, Figure 2A), and no evidence of heterogeneity was observed (I^2^ = 0.0%; P = 0.959). The findings of the dose–response meta-analysis also suggested no association between the risk of prostate cancer and a 100 μg/day increment of dietary folate intake (RR = 1.01; 95% CI = 0.99–1.02; P = 0.433, [I^2^ = 0.0%; P = 0.784], Figure 2B). As a result, a sensitivity analysis was conducted, and after each study was sequentially excluded from the pooled analysis, the conclusion was not affected by exclusion of any specific study.All studies were included in the dose–response curve between dietary folate intake and the incidence of prostate cancer. As shown in Figure 3A and illustrated by the P value for nonlinearity (P = 0.012), we found evidence of nonlinear relationships between dietary folate intake and the risk of prostate cancer. Dietary folate intake of more than 300 μg per day appeared to be associated with a non-significant reduction in the risk of prostate cancer.

### Serum folate levels and the risk of prostate cancer

**Figure 4:**
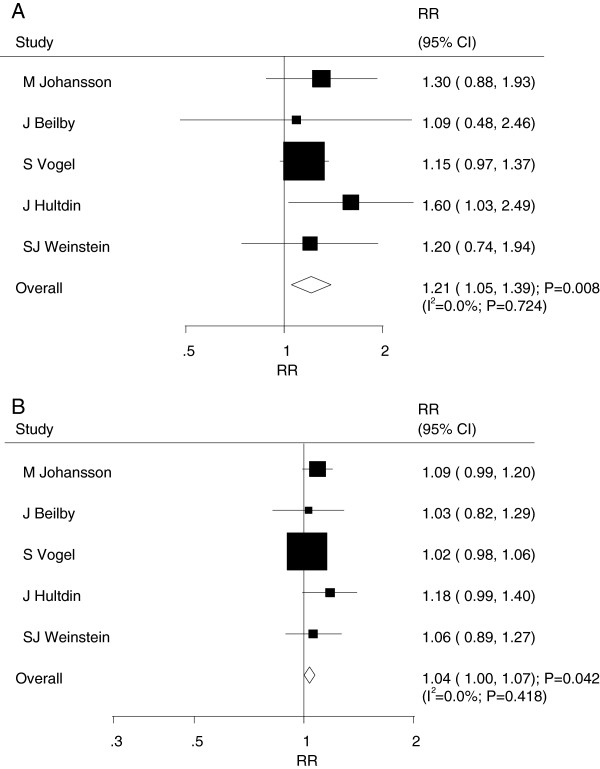
**Relative risk estimates of prostate cancer for high versus low serum folate levels (A) and per 5 nmol/L increment in serum folate levels for prostate cancer (B).**

A total of 5 prospective nest case control studies [7, 13–16] reported an association between serum folate levels and the risk of prostate cancer. The pooled analysis results for prostate cancer incidence indicated that the comparison of the high versus low categories of serum folate levels was associated with a harmful effect (RR = 1.21; 95% CI = 1.05–1.39; P = 0.008, with no evidence of heterogeneity [I^2^ = 0.0%; P = 0.724]; Figure 4A). The dose–response meta-analysis suggested that a 5 nmol/L increment of serum folate levels was associated with increased risk of prostate cancer (RR = 1.04; 95% CI = 1.00–1.07; P = 0.042, with no evidence of heterogeneity [I^2^ = 0.0%; P = 0.418], Figure 4B). Furthermore, as shown by the P value of nonlinearity (P = 0.111), there was no evidence of a potential non-linear relationship (Figure 3B).

### Subgroup analysis

Heterogeneity testing for the analysis identified a P value >0.10 for prostate cancer incidence. We concluded that heterogeneity is not significant in the overall analysis, suggesting that most variation was attributable to chance alone. Subgroup analyses were conducted to evaluate the effect of folate on prostate cancer risk in a specific population. Overall, we noted that a 5 nmol/L increment of serum folate levels was associated with the increased risk of prostate cancer if the duration of the follow-up less than 15 years. No other significant differences in effects were detected between dietary folate intake or serum folate levels and the risk of prostate cancer (Table 2).

**Table 2:** Subgroup analysis of risk ratios per 100 ug/day increase in dietary folate intake and per 5 nmol/L increase in serum folate levels for prostate cancer

Cancer sites	Group	RR and 95% CI	P value	Heterogeneity (%)	P value for heterogeneity
Dietary folate intake	Country				
	Europe	1.00 (0.98-1.02)	0.920	0.0	0.692
	Other	1.01 (0.99-1.03)	0.232	0.0	0.677
	Effect estimate				
	RR	1.00 (0.98-1.02)	0.743	0.0	0.361
	HR	1.01 (0.99-1.03)	0.421	0.0	0.682
	Follow-up (year)				
	15 or greater	1.01 (0.99-1.03)	0.421	0.0	0.682
	<15	1.00 (0.98-1.02)	0.743	0.0	0.361
	Adjusted age				
	Yes	1.01 (0.99-1.02)	0.601	0.0	0.577
	No	1.01 (0.98-1.03)	0.548	0.0	0.432
	Adjusted BMI				
	Yes	1.01 (0.98-1.03)	0.548	0.0	0.432
	No	1.01 (0.99-1.02)	0.601	0.0	0.577
	Adjusted alcohol consumption				
	Yes	1.01 (0.98-1.03)	0.548	0.0	0.432
	No	1.01 (0.99-1.02)	0.601	0.0	0.577
Serum folate levels	Country				
	Europe	1.05 (1.00-1.11)	0.062	23.2	0.272
	Other	1.03 (0.82-1.29)	0.798	-	-
	Effect estimate				
	RR	1.09 (0.99-1.20)	0.079	-	-
	OR	1.03 (0.99-1.07)	0.132	0.0	0.440
	Follow-up (year)				
	15 or greater	1.02 (0.98-1.06)	0.323	-	-
	<15	1.09 (1.02-1.18)	0.015	0.0	0.769
	Adjusted age				
	Yes	1.03 (0.82-1.29)	0.798	-	-
	No	1.05 (1.00-1.11)	0.062	23.2	0.272
	Adjusted BMI				
	Yes	1.06 (0.99-1.14)	0.102	48.0	0.146
	No	1.05 (0.91-1.21)	0.507	0.0	0.845
	Adjusted smoking				
	Yes	1.06 (0.99-1.14)	0.102	48.0	0.146
	No	1.05 (0.91-1.21)	0.507	0.0	0.845

### Publication bias

**Figure 5:**
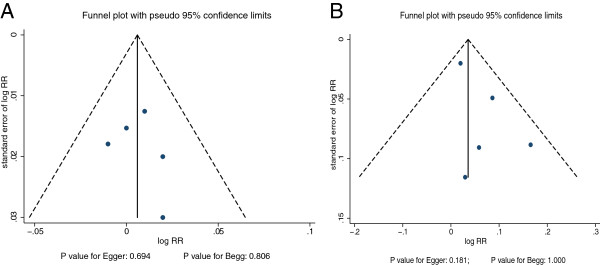
**Funnel plot for per 100 ug/day increment in dietary folate intake (A) and per 5 nmol/L increment in serum folate levels for prostate cancer (B).**

A review of funnel plots could not eliminate the potential for publication bias for prostate cancer (Figure 5). The Egger [28] and Begg test [29] results disclosed no evidence of publication bias for prostate cancer (Egger: P = 0.694 for dietary folate intake and P = 0.181 for serum folate levels; Begg: P = 0.806 for dietary folate intake and P = 1.000 for serum folate levels; Figure 5).

## Discussion

This study incorporated 10 available published prospective observational studies and provided a quantitative estimate of the association between dietary folate intake or serum folate levels and prostate cancer risk. After integrating all of the available evidence, we found that increased serum folate levels are associated with an increased risk of prostate cancer; furthermore, increased dietary folate intake has no significant effect on the risk of prostate cancer.

Although case control studies could give more information than most cohort studies concerning relatively short latency periods [30], we restricted our analysis to prospective observational studies (cohort studies and nested case–control studies) and excluded traditional case control studies, which are prone to recall and interviewer bias [30]. Furthermore, we assessed the methodological quality of the included studies using the NOS system [19] and evaluated the effect of folate levels on prostate cancer risk in specific populations in subgroup analyses to provide the best evidence for a potential relationship.

Of the 10 studies examined, the majority identified no association between dietary folate intake or serum folate levels and prostate cancer incidence, but 1 study [7] reported conflicting results. Hultdin et al. [7] demonstrated a significant effect for the higher serum folate level on the risk of prostate cancer. The pooled results of our meta-analysis was consistent with Hultdin’s study [7] and suggested that increased serum folate levels were associated with increased risk of prostate cancer, whereas increased dietary folate intake has no significant effect on prostate cancer risk. Furthermore, we discovered that the effect estimates of most studies exceeded 1 and revealed a potential trend of an adverse effect of increased dietary folate intake. We suggest there might be a harmful effect of dietary folate intake on the incidence of prostate cancer; however, this trend may not be obvious, and it should be validated by further research.

Most studies examining folate intake or serum folate status did not identify an association with prostate cancer risk [31, 32], although some previous prospective studies reported significant inverse associations of serum folate with prostate cancer incidence and mortality, but these associations between serum folate levels and prostate cancer risk could be modified by alcohol intake [33, 34]. Furthermore, it must be noted that relatively few events of cancer were reported, contributing to a low statistical power.

In our current study, there was no significant association between increased dietary folate intake and the risk of prostate cancer. The degree of association may be too small to detect an expected effect. Two possible explanations are that (1) different cooking methods may moderate the effect of dietary folate intake and (2) different types of prostate cancer might provide a biased view of the study question. Previous studies^31^ illustrated that increased folate intake is associated with only advance prostate cancer; however, data on prostate cancer type were not available, and thus, we could not differentiate the effects of dietary folate intake and serum folate levels by prostate cancer type.

Subgroup analyses indicated that the harmful effect of increased serum folate levels was more evident in studies with follow-up periods of less than 15 years than in those with longer follow-up periods. The reason for this difference could be that studies with longer follow-up periods (greater than 15 years) did not reach statistical significance owing to the low incidence of prostate cancer. This conclusion may be unreliable because smaller cohorts were included in each subset. Similar explanations may apply to subgroup analyses based on other potential biases factors.

Two strengths of our study compared with previous meta-analyses should be highlighted. First, only prospective studies were included, which should eliminate selection and recall bias. Second, the dose–response analysis included a wide range of dietary folate intake levels and statuses, which permitted an accurate assessment of the dose relationship between dietary folate intake or status and prostate cancer risk.

The limitations of our study are as follows: (1) publication bias is possible in meta-analyses of published studies; (2) data on prostate cancer type were not available, and thus, we could not differentiate the effects of dietary folate intake or status by prostate cancer type; and (3) the analysis used pooled data (individual data were not available), which restricted us from performing a more detailed relevant analysis and obtaining more comprehensive results.

## Conclusions

In conclusion, the findings of this study suggest that increased dietary folate intake was not associated with prostate cancer risk. However, increased serum folate levels were associated with an increased risk of prostate cancer. These findings need to be confirmed in future studies. Future studies should (1) ascertain the specific type of prostate cancer and analyse effects by type, (2) consider the effects of the cooking method when assessing dietary folate intake and its effects on clinical outcomes, and (3) consider the effects of alcohol consumption and smoking status.

## Electronic supplementary material

Additional file 1: **Checklist S1.** PRISMA Checklist. (DOC 70 KB)

Additional file 2: Table S1: Quality scores of prospective cohort studies using Newcastle-Ottawa Scale. (DOC 44 KB)
