# Supplementary material for: Folate intake, serum folate levels, and prostate cancer risk: a meta-analysis of prospective studies
Source: BMC Public Health. 2014 Dec 29;14:1326. doi: 10.1186/1471-2458-14-1326 (PMC4320532; doi:10.1186/1471-2458-14-1326)
Supplement: Supplementary file 2 — Additional file 2: Table S1: Quality scores of prospective cohort studies using Newcastle-Ottawa Scale. (DOC 44 KB) [file 12889_2014_7463_MOESM2_ESM.doc]

Table S1. Quality scores of prospective cohort studies using Newcastle-Ottawa Scale.

| Study | Selection | | | | Comparability | Outcome | | | NOS |
| --- | --- | --- | --- | --- | --- | --- | --- | --- | --- |
|  | Representativeness of the exposed cohort | Selection of the non exposed cohort | Ascertainment  of dietary/serum folate level | Demonstration that outcomes was not present at start of study | Comparability on the basis of the design or analysis | Assessment of outcome | Adequate follow-up duration | Adequate follow-up rate | Overall score |
| SJ Weinstein [8] 2006 | 1 | 1 | 1 | 1 | 2 | 1 | 1 | 1 | 9 |
| VL Stevens [9] 2006 | 1 | 1 | 1 | 1 | 2 | 1 | 0 | 1 | 8 |
| JK Bassett [10] 2012 | 1 | 1 | 1 | 1 | 2 | 1 | 1 | 1 | 9 |
| BAJ Verhage [11] 2012 | 1 | 1 | 1 | 1 | 1 | 1 | 1 | 1 | 8 |
| N Roswall [12] 2013 | 1 | 1 | 1 | 1 | 1 | 1 | 1 | 1 | 8 |
| M Johansson [13] 2008 | 1 | 1 | 1 | 1 | 1 | 1 | 0 | 1 | 7 |
| J Beilby [14] 2010 | 1 | 1 | 0 | 1 | 1 | 1 | 0 | 1 | 6 |
| S Vogel [15] 2013 | 1 | 1 | 1 | 1 | 2 | 1 | 1 | 1 | 9 |
| J Hultdin [7] 2005 | 1 | 1 | 1 | 1 | 2 | 1 | 0 | 1 | 8 |
| SJ Weinstein [16] 2003 | 1 | 1 | 1 | 1 | 1 | 1 | 0 | 1 | 7 |
